# Managing an effective treatment for neuroleptic malignant syndrome

**DOI:** 10.1186/cc5148

**Published:** 2007-01-12

**Authors:** Udo Reulbach, Carmen Dütsch, Teresa Biermann, Wolfgang Sperling, Norbert Thuerauf, Johannes Kornhuber, Stefan Bleich

**Affiliations:** 1 Department of Psychiatry and Psychotherapy, Friedrich Alexander University of Erlangen-Nuremberg, Schwabachanlage 6, D-91054 Erlangen, Germany

## Abstract

**Introduction:**

Neuroleptic malignant syndrome (NMS) is a rare, but sometimes fatal, adverse reaction to neuroleptics characterized principally by fever and rigor. The aim of this study was to prove the efficacy of different NMS treatment strategies, focusing on the efficacy of dantrolene.

**Methods:**

Altogether, 271 case reports were included. These cases were categorized into four treatment groups and compared to each other according to effectiveness of therapy within 24 hours, mortality, complete time of remission in days, effectiveness due to increase of dosage, relapse on the basis of decrease of dosage, and improvement of symptoms.

**Results:**

Between the four treatment groups, the complete time of remission was significantly different (analysis of variance, F = 4.02; degrees of freedom = 3; *p *= 0.008). In a logistic regression with adjustment for age, gender, and severity code, no significant predictor of the treatment for the complete time of remission (dichotomized by median) could be found. However, if the premedication was a monotherapy with neuroleptics, the complete time of remission was significantly shorter with dantrolene monotherapy (t = -2.97; *p *= 0.004).

**Conclusion:**

The treatment of NMS with drugs that are combined with dantrolene is associated with a prolongation of clinical recovery. Furthermore, treatment of NMS with dantrolene as monotherapy seems to be associated with a higher overall mortality. Therefore, dantrolene does not seem to be the evidence-based treatment of choice in cases of NMS but might be useful if premedication consisted of a neuroleptic monotherapy.

## Introduction

Neuroleptic malignant syndrome (NMS) is a rare, but sometimes fatal, adverse reaction to neuroleptics. It is characterized principally by fever and muscle rigidity. Furthermore, signs such as altered consciousness, autonomic instability, and laboratory findings such as elevated creatine phosphokinase (CPK), leukocytosis, raised liver enzymes, and low serum iron or potassium levels are also found (serum iron and potassium levels differ in the sets of diagnostic criteria according to several authors [1,2]). NMS is observed mainly in patients treated with neuroleptics, especially with high-potency neuroleptics, atypical neuroleptics, low-potency D2-receptor antagonists such as metoclopramide and tricyclic antidepressants, or after withdrawal of antiparkinsonians. Because clinical NMS studies have been conducted mainly in psychiatric units, it has been suggested that special attention to cancer patients undergoing psychopharmacologic treatment is necessary even in oncologic practice [3].

Although the origin of NMS remains unknown, a reduction in dopaminergic activity in the brain, probably by dopamine D2-receptor blockade in the striatum and hypothalamus, is generally assumed as its cause [4,5]. Nevertheless, other theories about its pathophysiology have been raised, including a disturbance of glutamate [6] or serotonin [7,8] receptors, central hyponatremia [9], overreaction of the sympathetic nerve system [8,10], as well as a kind of acute-phase reaction [11]. However, these theories are able to explain neither the symptoms mentioned above nor the low incidence rate between less than 0.1% and 2.5% of all patients treated with neuroleptic medication [12].

Therefore, a therapeutic approach inevitably seems to be through trial rather than evidence-based. It is generally agreed that it is of highest importance to identify the syndrome, suddenly withdraw the offending agent, and entertain supportive therapy as rehydration and restoring electrolyte balance. Because anticholinergics anticipate diaphoresis [11], they should be avoided. In regard to special treatment, several therapeutic options have been established. In addition to bromocriptine and other dopamine D2-receptor agonists, dantrolene sodium has been recommended predominantly in the past. Dantrolene is a peripheral muscle relaxant, which inhibits the intracellular calcium release from the sarcoplasmatic reticulum. It was originally applied to treat cases of malignant hyperthermia. It was first mentioned in 1981 as a treatment of NMS [12] and since then has been employed with more or less success. Nevertheless, dantrolene therapy is still considered the treatment of first choice in current pharmacologic and psychiatric textbooks as well as in recent publications [4,13]. Some authors have suggested a positive central effect of dantrolene in cases of NMS [14,15]. Since the mid-1980s, the benefit of specific treatment with dantrolene has been controversial [1]. No significant benefit or even a shortened course of recovery from NMS through single dantrolene therapy, compared to supportive therapy alone, could be observed [13,16]. In fact, failures of dantrolene therapy have been documented in several case reports [17-19]. as well as in relatively large samples [2].

Taking into account the low incidence rate of NMS, a randomized, controlled, and double-blinded prospective study did not seem to be feasible. Therefore, the aim of this study was to prove the efficacy of dantrolene therapy by a review of published cases and a complete review of the literature.

## Materials and methods

For facility of data recall, databases such as PubMed were searched to obtain a list of more than 600 publications from the years 1968 to 2006. Inclusion criteria for our study were the mention of therapy, treatment, dantrolene, case report, review of literature, and NMS since 1980 in title or abstract. Exclusion criteria were the exclusive mention of risk factors, pathophysiology, incidences and biochemistry, differential diagnosis, or foreign-language articles except those in German or English.

Altogether, 271 case reports including information on age, gender, diagnosis, and some data on therapy could be extracted. To avoid biases by multiply recorded case reports, case series were excluded as well. Information was registered as follows: year of publication, gender, age, diagnosis, triggering medications for NMS, dosages, time of incidence of NMS, fever, diaphoresis, pulse, rigidity, 'others' (blood pressure, level of consciousness, urinary retention, and so on), CPK, leukocytosis, other laboratory parameters (electrolytes, result of a lumbar puncture, and so on), time of withdrawal of the offending agent, dantrolene therapy (including dosage), adjuvant treatments such as cooling or others, course of illness, and time until complete recovery or death.

**Figure 1:**
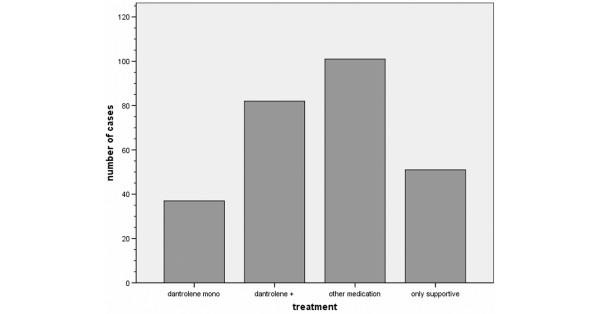
Distribution of frequency of neuroleptic malignant syndrome treatment. Dantrolene mono: dantrolene monotherapy; dantrolene +: dantrolene with additive medication (including bromocriptine, amantadine, and electroconvulsive therapy treatment); other medication: any medical therapy (excluding dantrolene); only supportive: cooling, infusion, and restoring electrolyte balance (no medication).

Because the main focus of the present study was to evaluate the efficacy of dantrolene and other treatments of NMS, cases were divided according to their received therapy into four treatment groups (Figure 1). The severity of NMS was assessed according to the Levenson criteria [1,4]. Thereby, it was possible to establish a comparable baseline status of patients within the aforementioned treatment groups.

Afterward, six categories pertaining to efficacy of treatment of NMS were assessed as follows: effectiveness of therapy within 24 hours, mortality, complete time of remission in days, effectiveness due to increase of dosage, relapse on the basis of decrease of dosage, and improvement of symptoms.

### Statistical methods

The complete time of remission was transformed by calculating the natural logarithm. To control for possible confounders, logistic models were performed in a second step. These models for the variables were adjusted for age, gender, and fever as a proxy measure for the severity of the NMS at baseline. Additionally, χ^2 ^tests and parametric (*t *tests, analysis of variance [ANOVA]) and nonparametric (Kruskal-Wallis) tests (in cases in which distribution was not normal and transformation was not reasonable) were calculated. The normal distribution of data was evaluated by the Kolmogorov-Smirnov test. All statistical tests were two-sided, and significance level was set at α = 0.05 or less. Data were analyzed using SPSS™ for Windows 11.0.1 (SPSS Inc., Chicago, IL, USA).

## Results

In summary, 271 case reports from 27 years (from 1980 to 2006) were included. Only 33.9% of the subjects included were female. The mean age of patients at the onset of NMS was 40.4 years (standard deviation 19.3). There was no significant difference in age between female and male patients (t = -0.87, *p *= 0.386).

Figure 1 shows the relative frequency of NMS treatment regimens in the present analysis.

The main criteria regarding effectiveness of therapy such as effectiveness of therapy within 24 hours, mortality, complete time of remission in days, effectiveness due to increase of dosage, relapse on the basis of decrease of dosage, and improvement of symptoms are displayed in Tables 1 and 2.

**Table 1:** Efficacy of treatment

	Effectiveness within 24 hours^a^		Complete remission in days	Mortality
		
	Yes	No		
Dantrolene monotherapy	23 (76.7%)	7 (23.3%)	9.4 (SD 12.7)	6/37 (16.2%)
Dantrolene with additive medication	30 (44.1%)	38 (55.9%)	19.0 (SD 31.6)	6/82 (7.3%)
Other medication	44 (67.7%)	21 (32.3%)	9.5 (SD 9.8)	9/101 (8.9%)
Only supportive therapy	9 (37.5%)	15 (62.5%)	9.2 (SD 18.4)	1/51 (2.0%)

^a^Missing values are due to the lack of detailed information in the reports. SD, standard deviation.

**Table 2:** Efficacy of treatment

	Effectiveness on the basis of increase of dosage	Relapse on the basis of decrease of dosage	Improvement of symptoms			
			
			Fever	Rigor	Both	Not mentioned
Dantrolene monotherapy	2/37 (5.4%)	3/37 (8.1%)	9 (24.3%)	4 (10.8%)	9 (24.3%)	15 (40.5%)
Dantrolene with additive medication	5/82 (6.1%)	7/82 (8.5%)	10 (12.2%)	3 (3.7%)	12 (14.6%)	57 (69.5%)
Other medication	4/101 (4.0%)	17/101 (16.8%)	12 (11.9%)	2 (2.0%)	14 (13.9%)	73 (72.3%)
Only supportive therapy	Not applicable	Not applicable	1 (2.0%)	-	1 (2.0%)	49 (96.1%)

Between treatment groups, significant differences in the effectiveness within 24 hours could be observed (χ^2 ^test: χ^2 ^= 16.0; degrees of freedom [df] = 3; *p *= 0.001). Interestingly, the short effectiveness of the dantrolene monotherapy was quite similar to other kinds of treatment, including bromocriptine, amantadine, or electroconvulsive therapy. Similar to the results of supportive therapy alone, the effectiveness of dantrolene including additive medication was weaker than in dantrolene as a monotherapy or other kinds of therapy regimens.

**Figure 2:**
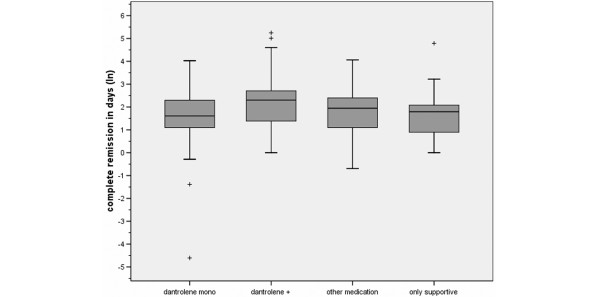
Complete remission in days categorized by treatment expressed through natural logarithm. To calculate the complete remission in days, the natural logarithm shown above should be multiplied by the Eularian constant (e). Shaded boxes: the box length is the interquartile range; plus signs: outliers: cases with more than 1.5 box lengths from the upper or lower edge of the box. Dantrolene mono: dantrolene monotherapy; dantrolene +: dantrolene with additive medication (including bromocriptine, amantadine, and electroconvulsive therapy treatment); other medication: any medical therapy (excluding dantrolene); only supportive: cooling, infusion, and restoring electrolyte balance (no medication).

As shown in Figure 2, the complete time of remission was significantly different between the four treatment groups (ANOVA, F = 4.02; df = 3; *p *= 0.008). After adjustment for multiple testing by the Bonferroni method, the complete time of remission was significantly shorter in only dantrolene monotherapy compared to dantrolene with additive medication (*p *= 0.012). In a logistic regression with adjustment for age, gender, and severity code, a significant predictor of the medicamentous treatment for the complete time of remission (dichotomized by median) could be found. In ascending order of elongated time of remission, the following odds ratios (ORs) were observed: 0.40 (95% confidence interval [CI] 0.20 to 1.19) for dantrolene monotherapy, 0.81 (95% CI 0.40 to 1.66) for a mainly supportive therapy, 1.06 (95% CI 0.59 to 1.90) for 'other medication,' and 1.56 (95% CI 0.84 to 2.91) for dantrolene with additive medication.

In regard to prior medicamentous treatment in addition to neuroleptics, lithium was administered in 30 (11.1%) cases and antidepressants in 15 (5.5%) cases. Overall, 114 (42.1%) cases of NMS were caused by neuroleptic monotherapies, 21 (7.7%) cases were caused by atypical neuroleptics, 17 (6.3%) cases were caused by depot/intramuscular application, and 16 (5.9%) cases were caused otherwise (for example, by withdrawal of antiparkinsonian agents). The remaining 96 (35.4%) cases were caused by other combination therapies (seven cases were described imprecisely in the case reports and were therefore excluded).

Furthermore, a significant association of prior medication with the complete time of regression could be observed (ANOVA, F = 2.76; df = 4; *p *= 0.029). Depot neuroleptics were found to have the highest complete time of remission, which was significantly longer than after NMS through monotherapy of typical neuroleptics, even after Bonferroni correction for multiple testing (*p *= 0.015).

In regard to the efficacy of dantrolene therapy, the history of medicamentous treatment prior to NMS was also relevant. If the premedication was a neuroleptic monotherapy, the complete time of remission was significantly shorter with a dantrolene monotherapy (t = -2.97; *p *= 0.004), whereas it was longer (if not significantly elongated) if premedication was comprised of a combination therapy of neuroleptics.

## Discussion

In regard to the efficacy of the dantrolene treatment, in our analysis the complete time of remission was prolonged by a combination with dantrolene treatment, and the mortality of a monotherapy was higher. Furthermore, the time of remission was not significantly shorter in a dantrolene monotherapy than in other therapy regimens including only supportive therapy. This has not been observed before; other studies [11,16,19]. did not examine the efficacy of dantrolene therapy in regard to monotherapy and therapy with additive medications. In our study, the additive treatment had the highest OR for an elongated complete regression time whereas the monotherapy showed the lowest OR. Considering the severity of the NMS at baseline, patients receiving dantrolene monotherapy were more severely ill than patients with other medications, which might explain the high mortality among the group of patients receiving dantrolene monotherapy.

A possible limitation of the present study is that it is based on case reports and some case reports were fragmentary in respect to the information necessary for our purposes. From the age of patients to dosage specifications, there was a wide range of missing information. Furthermore, none of the analyzed case studies addressed a suitable scale for the assessment of rigidity. Also, the temporal sequence of symptoms of NMS was not described by the latitude of studies.

The short time effectiveness within 24 hours was as effective in the dantrolene monotherapy group as in the group receiving different medication and was as ineffective in the dantrolene treatment group with additive medication as in the group of patients receiving supportive therapy alone. Nevertheless, in respect to the effectiveness due to increasing dosages, the relapse based on the decrease in dosage, or the improvement of symptoms such as fever and muscle rigidity, no obvious differences could be detected in our analysis.

Due to the different severity grades of NMS at baseline, mortality alone, which was examined as a predictor of the benefit of various medical treatments [19], does not seem to be sufficient to draw conclusions about the efficacy of each treatment. Therefore, other important variables such as effectiveness within 24 hours and complete time of remission were also examined.

Based on the findings of our analysis, dantrolene does not seem to be the evidence-based treatment of choice in cases of NMS, which seems to be in accordance with some single-case reports [2,20]. Nevertheless, there is also no evidence to explicitly deny a benefit of a dantrolene therapy. In some cases, dantrolene was very successfully used after some other vain treatment trials or after a period of time with only supportive treatment [21-23]. The latter might also be contradictory to the argument that time by itself leads to improvement [24].

Further investigations are still needed to discover both the etiopathology of NMS and its causal treatment. A promising approach might be the further exploration of possible central effects of dantrolene, which is still known as a peripheral muscle relaxant [12,13].

However, due to the low incidence of NMS, large prospective studies will be difficult to conduct, so further investigations will likely have to rely on case reports again. The success of such analyses most likely will depend on the accuracy, uniformity, and completeness of these reports.

Pharmacological risk factors for the development of NMS include high neuroleptic dosage, high rate of dose increase, and parenteral administration [25]. In regard to clinical risk factors, the available information is limited. It has been suggested that the recognition of an acute catatonic or disorganized syndrome is important in preventing NMS [26].

## Conclusion

Independent of treatment options, it is still necessary to discontinue potentially contributing medication even before the diagnosis is definite [27]. Dantrolene might not be the evidence-based treatment of choice in cases of NMS, but it might be useful if premedication consisted of a neuroleptic monotherapy.

## Key messages

• It is wise to discontinue contributing medication even before the diagnosis of NMS is definite.

• No treatment regimen including dantrolene could be considered as evidence-based.

## Abbreviations

ANOVA = analysis of variance; CI = confidence interval; CPK = creatine phosphokinase; df = degrees of freedom; NMS = neuroleptic malignant syndrome; OR = odds ratio.

## Competing interests

The authors declare that they have no competing interests.

## Authors' contributions

UR, JK, and SB made substantial contributions to the conception and design and to the analysis and interpretation of the data. CD made substantial contributions to the acquisition of data. TB, WS, and NT made substantial contributions to the interpretation of data. UR, CD, TB, and SB were involved in drafting the manuscript. WS, NT, and JK were involved in revising the manuscript critically for important intellectual content. All authors read and approved the final manuscript.
